# Greatly Enhancing Catalytic Activity of Graphene by Doping the Underlying Metal Substrate

**DOI:** 10.1038/srep12058

**Published:** 2015-07-09

**Authors:** Na Guo, Yongjie Xi, Shuanglong Liu, Chun Zhang

**Affiliations:** 1 Department of Physics and Graphene Research Centre, National University of Singapore, Singapore 117542; 2 Department of Chemistry, National University of Singapore, Singapore 117542.

## Abstract

Graphene-based solid-state catalysis represents a new direction in applications of graphene and has attracted a lot of interests recently. However, the difficulty in fine control and large-scale production of previously proposed graphene catalysts greatly limits their industrial applications. Here we present a novel way to enhance the catalytic activity of graphene, which is highly efficient yet easy to fabricate and control. By first-principles calculations, we show that when the underlying metal substrate is doped with impurities, the catalytic activity of the supported graphene can be drastically enhanced. Graphene supported on a Fe/Ni(111) surface is chosen as a model catalyst, and the chemical reaction of CO oxidation is used to probe the catalytic activity of graphene. When the underlying Fe/Ni(111) substrate is impurity free, the graphene is catalytically inactive. When a Zn atom is doped into the substrate, the catalytic activity of the supported graphene is greatly enhanced, and the reaction barrier of the catalyzed CO oxidation is reduced to less than 0.5 *eV.* Intriguing reaction mechanism of catalyzed CO oxidation is revealed. These studies suggest a new class of graphene-based catalysts and pave the way for future applications of graphene in solid-state catalysis.

Graphene has attracted enormous interests due to its unusual electronic properties originating from the unique honey-comb lattice^1^,^2^,^3^,^4^. Great research effort has been made in the last decade to potential applications of graphene as an electronic material. Very recently, a new direction in applications of graphene, graphene-based solid-state catalysis, started to attract attention^5^,^6^,^7^,^8^,^9^,^10^,^11^,^12^,^13^,^14^,^15^,^16^. Due to its chemical inertness, pristine graphene (in solid-state form) is not a good candidate for catalysts. Many ways have been proposed in literature to modify and activate graphene for supported metal catalysts such as applying a moderate mechanical strain^5^, introducing carbon-vacancy defects^6^, and doping impurity elements into graphene^7^,^8^,^9^,^10^. Some groups have demonstrated that inert graphene can be used as a cover to promote and ‘protect’ catalyzed chemical reactions^11^,^12^,^13^. Unusual effects of graphene on catalyzed chemical reactions have been reported in these studies. However, the difficulty in fine control and large-scale production of previously proposed graphene- supported or covered catalysts limits their industrial applications. To find a way that is efficient yet easy to fabricate and control to activate graphene is therefore essential for the future development of graphene-based solid-state catalysts.

Here through first-principles calculations we report a novel way of activating graphene for catalyzed CO oxidation. We show that when supported on a metal substrate that is doped with an impurity metal atom, graphene can be activated to be an excellent catalyst for the chemical reaction of CO oxidation. The observed high catalytic activity of graphene originates from the significant changes of local chemical properties of graphene right on top of the impurity due to charge transfer. Unlike previously suggested catalysts^5^,^6^,^7^,^8^,^9^ for which graphene serves as a substrate or a ‘cover’ for metal catalysts, in the novel catalysts proposed here, graphene itself is the catalytic center. Intriguing reaction mechanism of the catalyzed CO oxidation that is different from previously reported ones is revealed and discussed.

## Results and Discussions

Ni (111) is regarded as an excellent metal substrate for graphene due to the very small lattice mismatch between them. Graphene supported on Ni (111) has been extensively studied both experimentally and theoretically^17^,^18^,^19^,^20^. It has been pointed out that the van der Waals (vdW) interactions play an essential role in theoretically reproducing the experimentally observed stable adsorption configuration of graphene on Ni (111)^19^,^20^. A very recent experiment demonstrated that one or two monolayers of Fe can be ‘inserted’ between graphene and Ni (111)^21^. The Fe layers were shown to adopt the same lattice structure of Ni (111) surface and the supported graphene remains flat (without the Moiré pattern) after the Fe insertion. Unlike the case of graphene on Ni, previous DFT calculations without vdW interactions yielded adsorption energies and configurations of graphene on Fe/Ni (111) that agree well with experiments^21^. Since the vdW type of adsorption is not desired for graphene catalysis, in current study, we choose the two monolayer of Fe on Ni (111) as the underlying substrate, and then study the catalytic activity of the supported graphene.

**Figure 1:**
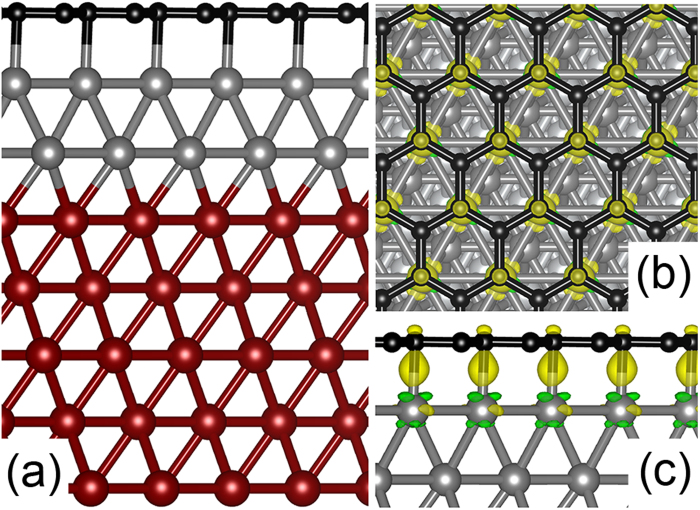
(**a**) Atomic model of graphene supported on Fe/Ni (111) (Ni in dark red, Fe in grey, C in black). Five layers of Ni are included in the model. The supercell includes 4 × 4 of pristine unit cell of graphene and 15 Å of vacuum in the direction perpendicular to the graphene surface; (**b**,**c**) are the top and side views of the isosurface of charge redistribution caused by the adsorption of graphene. The isosurface value of 0.008 e/Å3. The accumulation (depletion) of electrons is denoted by yellow (green).

The atomic model of graphene on Fe/Ni (111) is shown in Fig. 1a where five layers of Ni are included in the supercell to make sure that the correct surface properties of Ni (111) can be obtained. In structure optimizations, three bottom layers of Ni are fixed to bulk structures. Our DFT calculations show that the Ni-Fe and graphene-Fe bond lengths are around 2.48 Å and 2.15 Å, respectively. The adsorption energy of graphene is estimated to be 0.42 *eV* per carbon pair. All of these results agree very well with previous studies^21^. In Fig. 1b,c, we plot the isosurface of charge redistribution caused by the graphene adsorption. The formation of chemical bonding between graphene and the top Fe layer can be clearly seen from the figures. Note that we also tested the effects of vdW interactions by the so-called DFT+D^22^ calculations. The DFT+D method gives unreasonably high adsorption energy of graphene (more than 1.27 eV per carbon pair). We therefore do not consider vdW interactions in the rest part of the paper.

**Figure 2:**
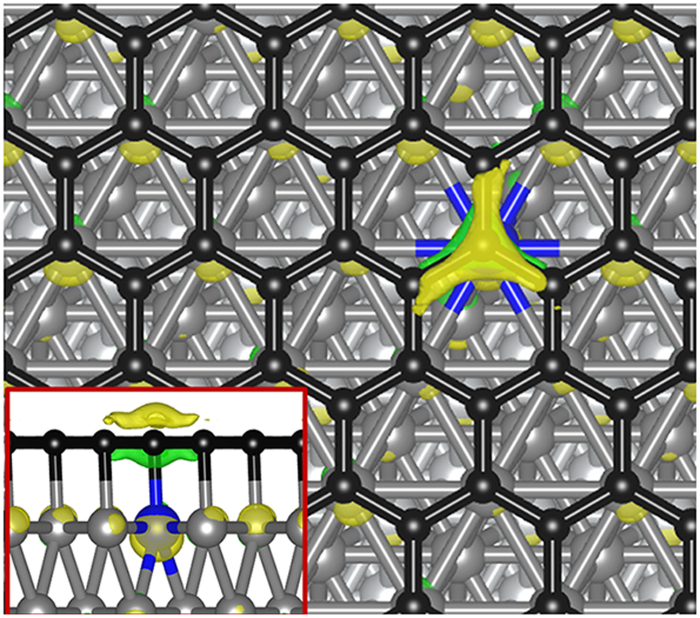
Graphene supported on Zn doped Fe/Ni (111). The Zn atom (in blue) is doped in the top Fe layer below the graphene. Superimposed we show the charge difference between electron densities with and without Zn doping. The color scheme is the same as Fig. 1. Inset: the side view of the charge difference.

The chemical reactivity of the supported graphene is tested by the adsorption of O_2_ and CO molecules. According to our calculations, both O_2_ and CO do not bind to graphene on impurity-free Fe/Ni (111), which is actually expected because of the chemical inertness of graphene. We then investigate the effects of substrate doping on properties of supported graphene. Compared with doping in graphene, the substrate doping we considered here has an obvious advantage that it does not ‘damage’ graphene and therefore is easier to control. We choose Zn as a dopant in current study because Zn is cost effective and has been widely used as dopant in various kinds of applications. One Zn atom is doped in the top Fe layer as shown in Fig. 2. After the Zn-doping, graphene remains essentially flat, while the local electronic properties of graphene significantly change. To demonstrate this, we superimpose in Fig. 2 the impurity-induced charge redistribution which is defined as the difference between electron densities with and without the impurity. The figure suggests that the Zn-C bond loses electrons compared with Fe-C bonds, and there are electrons accumulated on the impurity atom and the graphene surface right on top of the impurity, which weaken the bonding between the C atom on top of the impurity and the metal surface and in turn change the local chemical reactivity. As a result, after the Zn doping, O_2_ does bind to graphene at places around the C atom that forms chemical bond with the impurity Zn atom.

**Figure 3:**
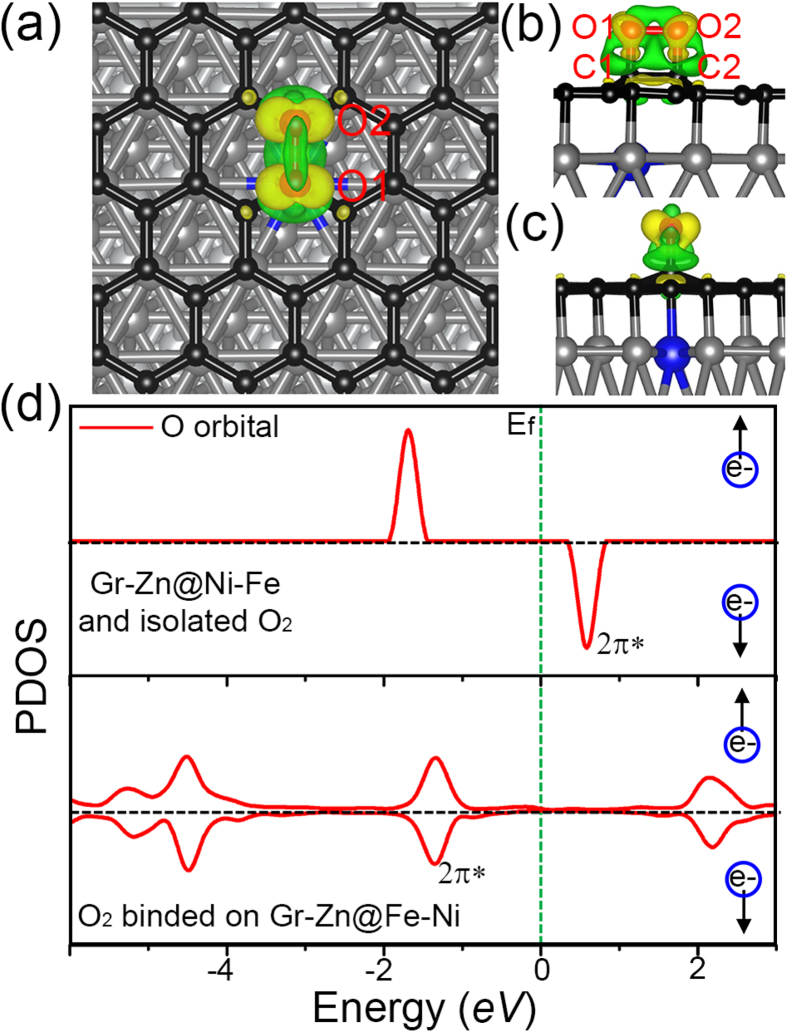
(**a**) O_2_ (O in red) adsorbed on graphene supported on Zn doped Fe/Ni (111). The adsorption energy of O_2_ is around 0.42 *eV*. The isosurface of charge redistribution caused by O_2_ adsorption is superimposed. (**b**,**c**) are side views of charge redistribution. (**d**) projected density of states (PDOS) of O_2_ molecule before (upper) and after (lower) adsorption. The PDOS of O_2_ before adsorption is calculated by putting the molecule 5 Å away from the graphene in the same supercell.

We show in Fig. 3a the most stable adsorption configuration of O_2_ on graphene that is supported on Zn doped Fe/Ni (111). The adsorption energy of O_2_ in this case is calculated to be around 0.42 *eV*. The O-O bond of the O_2_ molecule is significantly elongated to 1.48 Å (compared with 1.24 Å in gas phase). We also tested other inequivalent adsorption sites near the Zn impurity (Figure S1 in the supporting information), and found that the site shown in Fig. 3a is the only one that shows chemisorption of O_2_. When far away from the impurity atom, there is no binding of O_2_. Note that CO still does not bind to graphene after doping. We also considered cases of doping multiple Zn atoms in the supercell and found that when two Zn impurities are close to each other, only one O_2_ is adsorbed on graphene (see Figure S2 in the supporting information). The adsorption energy O_2_ is calculated from equation, 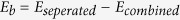, where 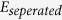 is the energy of the supported graphene and an O_2_ that is 5 Å away from graphene surface.

**Figure 4:**
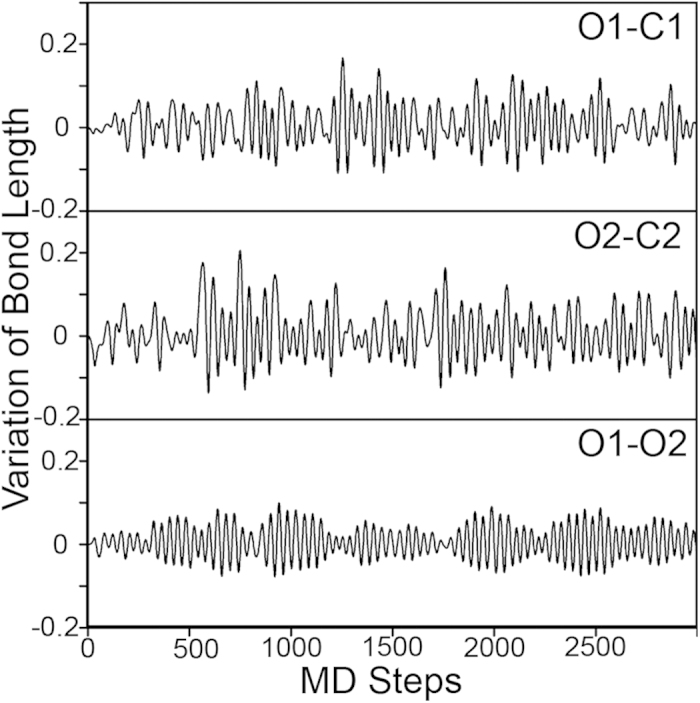
Variations of bond lengths (in Å) as functions of MD steps. The MD simulations were done for O_2_ adsorbed on graphene-Zn@Fe/Ni (111). O1, O2, C1, C2 are atoms as shown in Fig. 3b. The temperature is set to 300 K. NVT ensemble is used in simulations. The MD step is set to 1 *fs*.

We would like to point out here that since the system under study is big, the numerical error in total energy calculations could be big also so that the calculation of the adsorption energy of O_2_ may not be accurate. To confirm the stability of the O_2_ adsorption, we conducted *ab initio* molecular dynamics (MD) simulation of the system with adsorbed O_2_ (Fig. 3a) at room temperature 300 K. The variations of bond lengths (the difference between the current length of the bond and its equilibrium value) of O-O (O_2_) and two C-O bonds (between graphene of O_2_) as functions of MD steps are shown in Fig. 4. The variation of O-O bond length at room temperature is always less than 0.1 Å, and the bond lengths of two C-O bonds oscillate between ±0.1 Å after 2000 MD steps, indicating that the adsorption of O_2_ on graphene after the impurity doping is stable at room temperature. To understand the interaction between the supported graphene and the O_2_ molecule, we plot the isosurface of charge redistribution caused by O_2_ adsorption in Fig. 3a. The side views of the charge redistribution are shown in Fig. 3b,c. It can be seen that electrons transferred to 2π^*^ orbital of O_2_, causing the O-O bond elongated. The adsorption induced change of O_2_ electronic structures is further illustrated in Fig. 3d where the projected density of states (PDOS) of O_2_ molecule before and after adsorption is plotted. Before adsorption, the O_2_ molecule is magnetic and the 2π^*^ orbital of spin-down channel is unoccupied. After adsorption on graphene, three are electrons transferred to the originally empty 2π^*^, pulling the orbital below the Fermi energy and eliminating the magnetic moment of the molecule. This picture is consistent with the Bader charge analysis that nearly one electron is transferred to O_2_ after adsorption.

**Figure 5:**
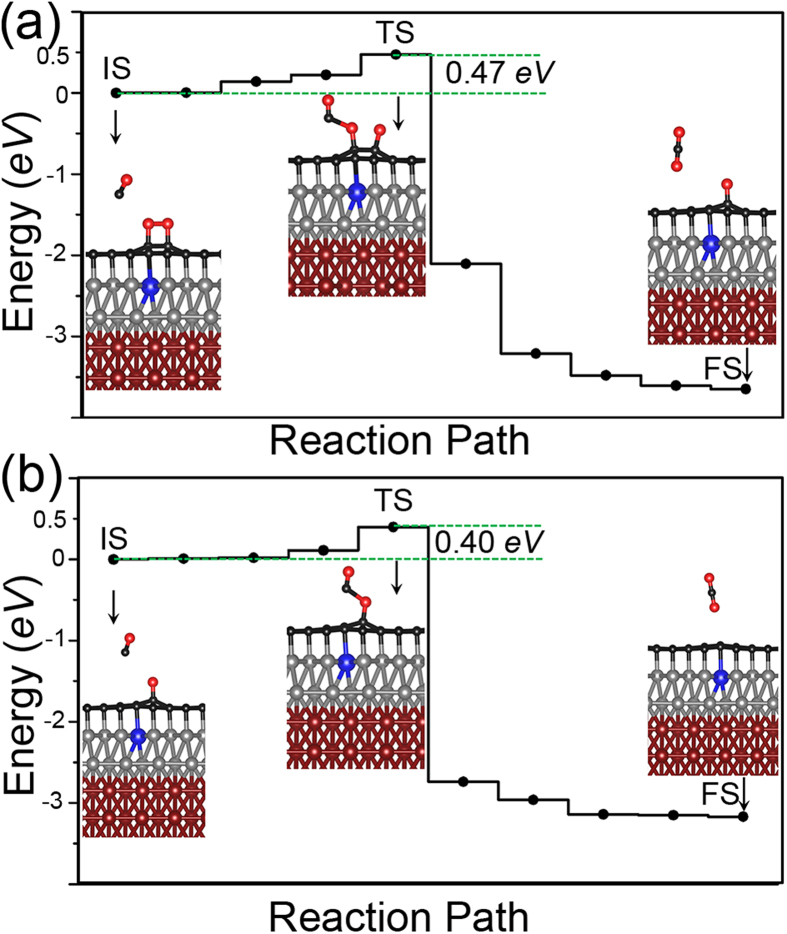
The energy profile of two-step CO oxidation catalyzed by graphene supported on Zn@Fe/Ni (111). The calculations are done by the c-NEB method. (**a**) the first step of the reaction: CO+O2→CO2+O. The initial state (IS): d(O-O) = 1.48 Å, d(C-O) = 1.14 Å, and d(CO-O_2_) = 3.10 Å; The transition state (TS): d(O-O) = 2.12 Å, d(C-O) = 1.16 Å, and d(CO-O_2_)=1.85 Å, in the final state (FS), CO_2_ forms. (**b**) the second step of the reaction: CO+O→CO2. The initial state (IS): d(C-O)=1.14 Å, and d(CO-O_2_) = 3.30 Å; The transition state (TS): d(C-O) = 1.17 Å, and d(CO-O) = 1.76 Å. CO_2_ does not bind to the graphene surface in both cases. Note that d(O-O) denotes the O-O bond length of O_2_, d(C-O) the C-O bond length of CO, and d(CO-O_2_) the distance between the C atom in CO and the nearest O in O_2_.

The significantly elongated O-O bond of the adsorbed O_2_ implies that the molecule is activated for CO oxidation. Since CO does not bind to graphene, there are no CO poisoning problems and we only need to consider the Eley-Rideal (ER) mechanism of the reaction for which the CO molecule approaches the O_2_ from vacuum. As aforementioned, the reaction barrier is determined by c-NEB method. In Fig. 5, we show the energy profile along the reaction paths for two steps of the CO oxidation in a whole cycle of the catalyzed reaction (the first step: CO+O_2_→CO_2_+O and the second step: CO+O→CO_2_). Reaction barriers of both steps are low (<0.5 *eV*), indicating high catalytic activity of the supported graphene. In the first step of the reaction, the barrier of 0.47 *eV* originates from the breaking of the O-O bond and also one C-O bond between the O_2_ and graphene. To get some rough ideas for how much these two bond breaking processes contribute to the barrier, we performed c-NEB calculations for the breaking of O-O bond on graphene (without the presence of the CO molecule). The results of calculations are shown in Figure S3 in the supporting information, from which we can see that around 0.31 *eV* of energy is needed to break the O-O bond on graphene surface. After one O atom of O_2_ is taken away in the first step of reaction, the binding between the left-over O atom and the graphene becomes stronger, which can be seen from the fact that in this case the C-O bond is shorten from 1.50 Å to 1.35 Å. The shorter C-O bond length means more energy is needed to break the bond. The reaction barrier of the second step of the reaction is estimated to be 0.40 *eV* (Fig. 5b). The barrier is mainly from the breaking of the left-over C-O bond. The barriers of two steps are similar, suggesting that two steps are equally important and the reaction rate of the catalyzed CO oxidation is determined by both steps.

## Conclusion

In summary, via *ab initio* calculations, we present a novel method of activating graphene for catalyzing chemical reaction of CO oxidation. When supported on impurity free Fe/Ni (111) surface, graphene is chemically inert. We show that after doping an impurity Zn atom in the underlying Fe layer, graphene is activated and become an excellent catalyst for CO oxidation. The reaction barriers of the two-step chemical reaction of CO oxidation catalyzed by graphene supported on Zn doped Fe/Ni (111) are estimated to be low (<0.5 *eV*). Unlike the previously proposed graphene- supported or covered catalysts, the catalysis center here is the graphene itself. Since CO does not bind to graphene, the CO poisoning is not an issue for the proposed catalyst here. Also, considering the cost-effective materials (Ni, Fe, Zn, C) we used here, the proposed method of activating graphene may have important applications in the future design of graphene-based catalysis. We believe that the observed substrate-impurity induced high catalytic activity of graphene is not limited to the specific system discussed in this paper. Our studies pave a way for a new class of graphene-based solid-state catalysts.

### Computational Methods

The first-principles calculations are performed with spin-polarized density functional theory (DFT) by utilizing Vienna ab-initio Simulation Package (VASP)^23^,^24^. Reaction barriers of catalyzed CO oxidations are determined by the Climbing Image Nudged Elastic Band (c-NEB) method^25^. The generalized gradient approximation (GGA) in the Perdew-Burke-Ernzerh (PBE) format^26^ and the projector-augmented wave (PAW) method^27^ are employed in all calculations. A plane wave basis with the cut-off energy of 450 *eV* and 3 × 3 × 3 sampling of the Brillouin zone are used. The convergence criterion for structural relaxations is set to 0.01 *eV/Å*.

## Additional Information

**How to cite this article**: Guo, N. *et al.* Greatly Enhancing Catalytic Activity of Graphene by Doping the Underlying Metal Substrate. *Sci. Rep.* **5**, 12058; doi: 10.1038/srep12058 (2015).

## Supplementary Material

Supporting Information
