# Supplementary material for: Greatly Enhancing Catalytic Activity of Graphene by Doping the Underlying Metal Substrate
Source: Sci Rep. 2015 Jul 9;5:12058. doi: 10.1038/srep12058 (PMC4496776; doi:10.1038/srep12058)
Supplement: Supporting Information [file srep12058-s1.pdf]

SUPPORTING INFORMATION

Greatly Enhancing Catalytic Activity of Graphene by Doping the Underlying Metal Substrate

Na Guo, [‡,†] Yongjie Xi, [‡,§,†] Shuanglong Liu,[†] and Chun Zhang*[,†,§]

[†]Department of Physics and Graphene Research Centre, National University of Singapore, Singapore 117542

[§]Department of Chemistry, National University of Singapore, Singapore 117542

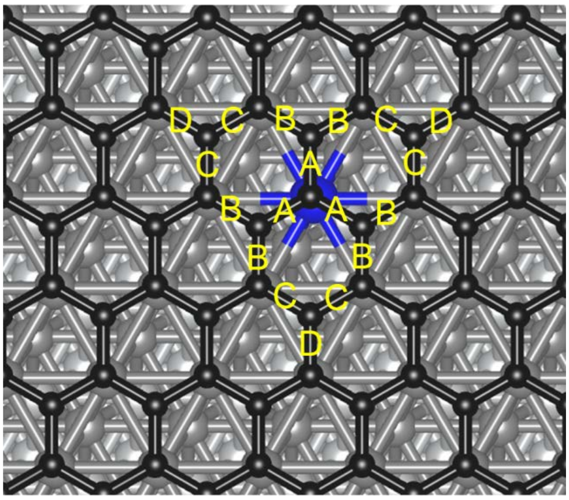

Figure S1. Possible adsorption sites of O2 on graphene-Zn@Fe/Ni (111). We tested four inequivalent sites near the impurity Zn (denoted as A, B, C, D in the figure). The calculated adsorption energies are 0.42 *eV* for site A, 0.02 *eV* for site B, and there are no binding of O2 on other sites.

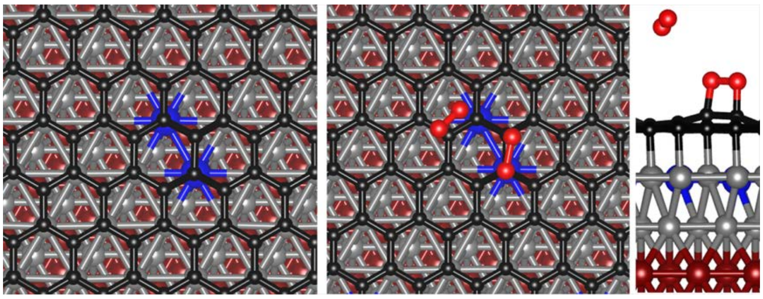

Figure S2. Left: Two Zn impurities are close to each other; Middle: The adsorption of two O_2 molecules on graphene-Zn@Fe/Ni (111). Right: Side view. Note that in this case, only one O_2 is adsorbed.

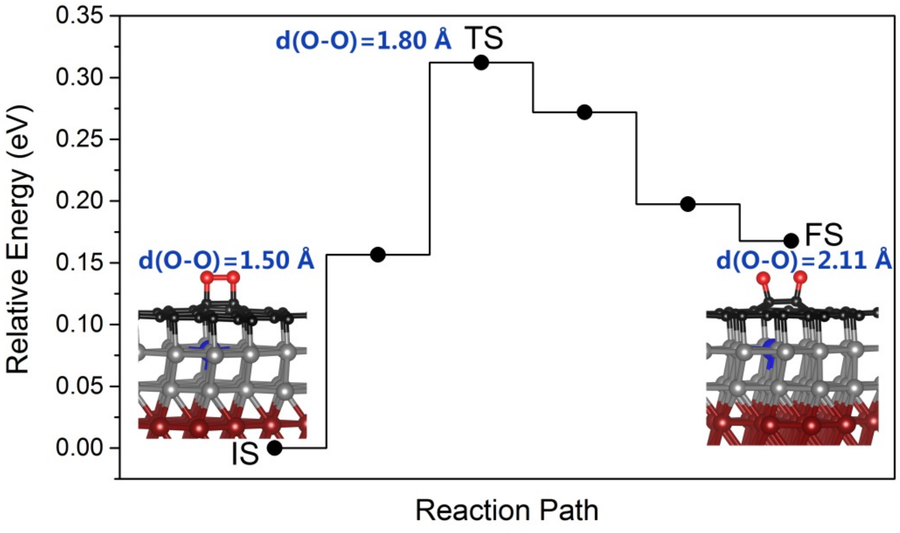

Figure S3. Energy profile of O_2 dissociation on graphene-Zn@Fe/Ni (111). Simulations were done using c-NEB method. It was found that about 0.31 *eV* is needed to break the O-O bond of O_2 adsorbed on graphene surface. Initial State (IS): O_2 adsorbed on graphene with a bond length of 1.50 Å. Transition State (TS): the O-O bond length is 1.80 Å. Final State (FS): O_2 dissociated on graphene.
